# Delineating the molecular landscape of different histopathological growth patterns in colorectal cancer liver metastases

**DOI:** 10.3389/fimmu.2022.1045329

**Published:** 2022-12-16

**Authors:** Mingtao Hu, Zhigang Chen, Dandan Hu, Shaoyan Xi, Deshen Wang, Xiaolong Zhang, William Pat Fong, Lei Wen, Yanyu Cai, Yunfei Yuan, Binkui Li, Xiaojun Wu, Zhenhai Lu, Gong Chen, Liren Li, Peirong Ding, Zhizhong Pan, Desen Wan, Ziming Du, Minshan Chen, Yuhong Li

**Affiliations:** ^1^ State Key Laboratory of Oncology in South China, Collaborative Innovation Center for Cancer Medicine, Sun Yat-Sen University Cancer Center, Guangzhou, China; ^2^ Department of Medical Oncology, Sun Yat-Sen University Cancer Center, Guangzhou, China; ^3^ Department of Hepatobiliary Surgery, Sun Yat-Sen University Cancer Center, Guangzhou, China; ^4^ Department of Pathology, Sun Yat-Sen University Cancer Center, Guangzhou, China; ^5^ Department of Colorectal Surgery, Sun Yat-Sen University Cancer Center, Guangzhou, China; ^6^ Department of Molecular Diagnostics, Sun Yat-Sen University Cancer Center, Guangzhou, China

**Keywords:** colorectal cancer liver metastases, histopathological growth pattern, RNA sequencing, tumor microenvironment, soil and seeds

## Abstract

**Background:**

Histopathological growth patterns (HGPs) have shown important prognostic values for patients with colorectal cancer liver metastases, but the potential molecular mechanisms remain largely unknown.

**Methods:**

We performed an exploratory analysis by conducting the RNA sequencing of primary colorectal lesions, colorectal liver metastatic lesions and normal liver tissues.

**Findings:**

We found that desmoplastic HGPs of the metastatic lesions were significantly enriched in EMT, angiogenesis, stroma, and immune signaling pathways, while replacement HGPs were enriched in metabolism, cell cycle, and DNA damage repair pathways. With the exception of immune-related genes, the differentially expressed genes of the two HGPs from colorectal liver metastases were mostly inherited from the primary tumor. Moreover, normal liver tissue in the desmoplastic HGP subgroup was markedly enriched in the fibrinous inflammation pathway.

**Conclusions:**

We surmised that HGPs are observable morphological changes resulting from the regulation of molecular expressions, which is the combined effect of the heterogeneity and remodeling of primary tumors seeds and liver soils.

## Introduction

Approximately 50% of patients with colorectal cancer develop liver metastases during the course of their disease, making it the leading cause of death in the majority of patients (1). Hepatectomy is the main curative treatment option for colorectal cancer liver metastasis (CRLM). However, over 50% of patients suffer from recurrence within 2 years (2). In recent years, histopathological growth patterns (HGPs) have attracted extensive attention as prognostic factors in lesions with positive resection margins, recurrent disease and overall survival following complete resection (3–5). The HGPs are defined due to the interface between the metastatic cancer cells and the surrounding normal liver cells examinating on standard hematoxylin & eosin-stained (H&E) tissue sections under the light microscopy, which are easily obtainable, inexpensive and reproducible (6). Actually, they are comprehensive parameters of tumor and surrounding tumor microenvironment (TME), including tumor invasion ability, angiogenesis, paracrine and autocrine effects of growth factors, fibrosis, and tumor immune microenvironment. These particular patterns have been previously described in liver metastases originating from colorectal cancer, gastric cancer, pancreatic cancer, uveal melanoma and cutaneous melanoma (7–11). In the CRLM, the desmoplastic HGP (dHGP, a rim of desmoplastic stroma separating the tumor cells and the normal liver parenchyma) and replacement HGP (rHGP, the tumor cells invade along the liver cell plates and replace normal hepatocytes) are the two most common HGP subtypes.

Accumulating studies have explored the pathological morphology heterogeneity between the two subtypes. Replacement and desmoplastic lesions have been shown to possess different forms of tumor vascularization (12). Replacement HGP can utilize vessel co-option rather than angiogenesis to create their blood supply, leading to resistance to anti-angiogenic therapy (13). The two subtypes also possess different tumor immune microenvironments. Our previous study showed that dHGP was correlated to a high immunoscore in pathological tissues and predicted a favorable prognosis independent of the immunoscore (14). However, the potential molecular mechanisms underlying HGPs remain largely unknown.

Thus, this study aimed to compare the different molecular signatures between the dHGP and rHGP subtypes at the RNA level. Furthermore, we explored how the distinctions between the “seed” (the primary tumor) and “soil” (the normal liver tissue) of those two HGP subtypes might contribute to the different HGP formations.

## Methods

### Sample collection

**Figure 1:**
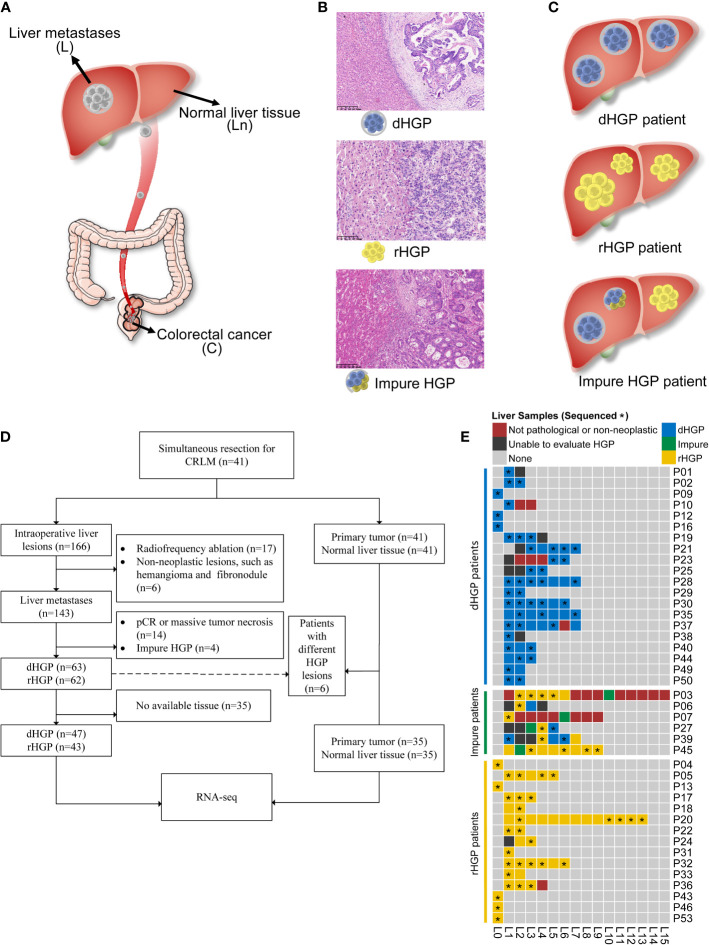
Study design. **(A)** Tissues were obtained from patients undergoing colectomy and hepatic resection in this study. **(B)** Evaluations of HGP were done for liver metastasis and each patient. **(C)** Patient enrolment and samples were included in the analyses. Of the 41 enrolled patients, a total of 90 liver metastasis lesions, 35 normal liver tissues, and 35 primary lesions were included in the transcriptome sequencing and analyses. **(D)** A heatmap depicting the HGP for each liver metastasis and each patient. **(E)** Pathological evaluation and RNA sequencing of all liver lesions. *: the sample was sent to RNA sequencing.

This study was approved by Sun Yat-sen University cancer center’s medical ethics committee and was performed according to the Helsinki Declaration of the World Medical Association. We collected fresh-frozen samples from a clinical cohort of patients who underwent simultaneous R0 resection for simultaneous colorectal liver metastases at the Sun Yat-sen University cancer center from July 2019 to September 2020. Histologically confirmed primary colorectal lesions (C) and metastatic liver lesions (L), as well as normal liver tissues as far away from tumors as possible (Ln), were collected (**Figure 1A**). Clinical characteristics, such as age, gender, tumour location and TNM stage, were collected retrospectively from medical records. Patients were followed up until July 2021. Relapse-free survival (RFS) was defined as the time from curative operation to the first relapse or last follow-up.

### Pathological assessment of HGPs

Each available H&E-stained and formalin-fixed paraffin-embedded (FFPE) liver lesion specimens were assessed in accordance with the international consensus guidelines by two independent pathologists using a light microscope (6). Examples for displaying the dHGP, rHGP, or mixed lesions are shown in **Figure 1B**. In this study, the liver lesion was categorized as dHGP or rHGP lesion if only dHGP or rHGP was present at the interface. The liver lesion with more than one HGP at the interface was categorized as an impure liver lesion. Patients presenting all dHGP or rHGP liver metastases were classified as the dHGP or rHGP patients, respectively, while the patients showing different HGP liver lesions or impure liver lesions were defined as impure HGP patients (**Figure 1C**).

Primary tumor and normal liver tissues from pure HGP patients, as well as all available liver lesions, were collected for RNA sequencing. In order to better compare the differences between dHGP liver metastases and rHGP liver metastases, only pure lesions were included. Similarly, only pure patients were included in the comparision for the primary tumor and normal liver tissue.

### Sample preparation, library construction, and RNA sequencing

RNA purity was verified using the NanoPhotometer^®^ spectrophotometer (IMPLEN, CA, USA), while RNA integrity was assessed using the RNA Nano 6000 Assay Kit of the Bioanalyzer 2100 system (Agilent Technologies, CA, USA). Sequencing libraries were generated using NEBNext^®^ UltraTM RNA Library Prep Kit for Illumina^®^ (NEB, USA). The library preparations were sequenced on an Illumina Hiseq platform, and 125 bp/150 bp paired-end reads were generated. In total, 160 samples, including 90 liver metastases, 35 normal liver tissues, and 35 primary lesions, were successfully sequenced using this approach.

### Statistical analysis

All statistical analyses were performed using R Statistical Software (version 3.6.3). The Chi-square tests were employed to examine the associations of categorical variables, while the Wilcoxon test was utilized to compare two groups. The pROC package for R was applied to calculate AUC to examine the predictive accuracy of each differentially expressed gene (DEG), C-score, and Ln-score for HGP type of liver metastases. The interactions were estimated by Spearman correlation analyses. The associations of HGP or risk score with RFS were examined by Kaplan-Meier and Cox proportional hazard analyses. And the log-rank test was employed. The stats package for R was applied to conduct principal component analysis (PCA). All the above calculations were done with TPM data. All statistical *p*-values were two-sided.

## Results

In total, 166 intraoperative liver lesions from 41 patients were found. 125 lesions were histologically confirmed metastatic colorectal adenocarcinoma and assessed as pure dHGP or rHGP according to the pathological examination. Six patients had different HGP liver lesions, and 35 patients were pure HGP (20 dHGP and 15 rHGP) (**Figures 1D, E**).

There were no significant differences in gender, age, pT stage and pN stage between the two groups. The primary tumor of colorectal cancer in both groups were mainly located in the left colon and rectum [14 patients (70.0%) vs. 10 patients (66.7%), P =0.833]. Most patients had received preoperative chemotherapy [18 (90.0%) vs. 13 (86.7%), P =0.759]. All three patients with viral hepatitis were dHGP, but there was no statistically significant difference [3 (15.0%) vs. 0 (0%), P =0.117] (**Table 1**).

**Table 1:** Clinical characteristics of dHGP and rHGP patients.

Clinical characteristics	dHGP patients (N = 20)	rHGP patients (N = 15)	P value
**Gender**			**0.829**
male	14 (70.0)	11 (73.3)	
Female	6 (30.0)	4 (26.7)	
**Age**			**0.398**
mean ± standard deviation	56.9 ± 8.1	54.2 ± 10.5	
**Location of primary tumor**			**0.833**
Left hemicolon and rectum	14 (70.0)	10 (66.7)	
Right hemicolon	6 (30.0)	5 (33.3)	
**pT stage**			**0.660**
T1-2	1 (5.0)	2 (13.3)	
T3	17 (85.0)	12 (80.0)	
T4	2 (10.0)	1 (6.7)	
**pN stage**			**0.127**
N0	12 (60.0)	5 (33.3)	
N1	7 (35.0)	6 (40.0)	
N2	1 (5.0)	4 (26.7)	
**Extrahepatic metastasis**			**0.393**
Yes	5 (25.0)	2 (13.3)	
No	15 (75.0)	13 (86.7)	
**Preoperative chemotherapy**			**0.759**
Yes	18 (90.0)	13 (86.7)	
No	2 (10.0)	2 (13.3)	
**Combined with bevacizumab**			**0.698**
Yes	3 (15.0)	3 (20.0)	
No	17 (85.0)	12 (80.0)	
**Combined with cetuximab**			**0.599**
Yes	7 (35.0)	4 (26.7)	
No	13 (65.0)	11 (73.3)	
**History of hepatitis B**			**0.117**
Yes	3 (15.0)	0 (0)	
No	17 (85.0)	15 (100)	
**Drinking History**			**0.833**
Yes	1 (5.0)	1 (6.7)	
No	19 (95.0)	14 (93.3)	
**Smoking history**			**0.167**
Yes	1 (5.0)	3 (20.0)	
No	19 (95.0)	12 (80.0)	
**History of Hypertension**			**0.265**
Yes	4 (20.0)	1 (6.7)	
No	16 (80.0)	14 (93.3)	
**History of diabetes**			**0.265**
Yes	4 (20.0)	1 (6.7)	
No	16 (80.0)	14 (93.3)	

The bold values are Clinical characteristics.

### Comparison of the transcriptome landscapes between the 47 dHGP and 43 rHGP liver metastatic lesions

**Figure 2:**
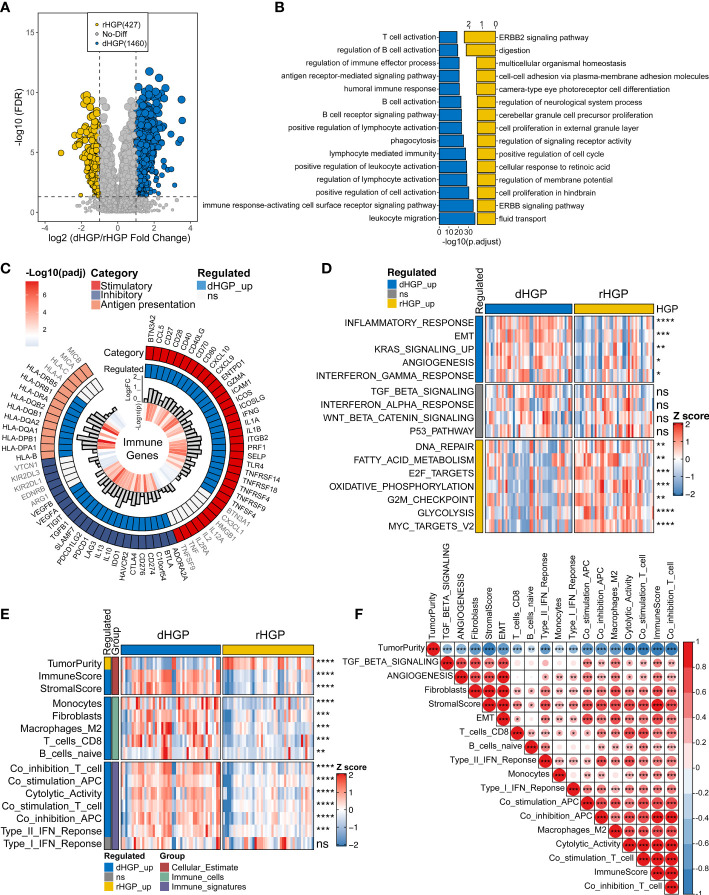
Comparison of the transcriptome landscapes between dHGP and rHGP liver metastases. **(A)** Volcano plot displaying 1460 up-regulated DEGs in the dHGP subgroup and 427 up-regulated DEGs in the rHGP subgroup (|LogFC| > 1, FDR < 0·05). **(B)** GO-BP enrichment analysis of the differentially expressed genes (FDR < 0·05). **(C)** Circos plot shows 55/72 up-regulated immune genes in the dHGP samples (LogFC > 0, FDR < 0·05). **(D)** Heatmap depicting the differences of HALLMARK pathways activity between dHGP and rHGP subgroups (Wilcoxon test. ns, *p* > 0·05; *p < 0·05; **p < 0·01; ***p < 0·001; ****p < 0.0001). Rows represent GSVA scores of HALLMARK pathways, and columns represent samples. **(E)** Heatmap depicting the differences between two subgroups in TIME-relevant molecular signatures were linked to cellular estimates, immune cells and immune signatures (Wilcoxon test. ns, *p* > 0·05; **p < 0·01; ***p < 0·001; ****p < 0.0001). Rows represent ssgsea scores of TIME-relevant molecular signatures and columns represent samples. **(F)** Correlations between selected HALLMARK pathways activity and TIME-relevant molecular signatures (Spearman correlation analysis. *p < 0·05; **p < 0·01; ***p < 0·001).

Differential expression analysis between 47 dHGP and 43 rHGP liver metastatic lesions was performed (|LogFC| > 1, false discover rate (FDR) < 0·05; **Table S2**; **Figure 2A**). The GO-BP enrichment analysis were conducted to identify the biological function of DEGs (FDR < 0·05, **Table S3**; **Figure 2B**). 1460 DEGs were up-regulated in the dHGP subgroup and were found to be markedly enriched in immune-related biological processes. Among them, T-cell activation was the most significantly enriched pathway. Of the 72 important immune genes, 55 were up-regulated in the dHGP liver metastases (FDR < 0·05; **Figure 2C**). Meanwhile, 427 DEGs were up-regulated in the rHGP subgroup and were mainly associated with cell proliferation and cell cycle, including the ERBB2 signaling pathway. Furthermore, comparisons of the HALLMARK pathways activity indicated that dHGP liver metastatic lesions were significantly enriched in specific cancer-related pathways, including epithelial-mesenchymal transition (EMT), angiogenesis, KRAS signaling pathway, inflammatory response, and interferon-gamma response, while the rHGP liver metastatic lesions were associated with DNA repair, fatty acid metabolism, E2F targets, oxidative phosphorylation, G2M checkpoints, glycolysis and MYC target (Wilcoxon test, *p* < 0·05; **Tables S4, S5**; **Figure 2D**). With a higher immune and stromal score, dHGP liver metastases were characterized by a higher abundance of monocytes, fibroblasts, M2 macrophages, CD8+ T cells, and naive B cells and obtained higher scores from 7 immune-related signatures, indicating that the TIME had a better anti-tumor ability (Wilcoxon test, *p* < 0·05; **Tables S6, S7**; **Figure 2E**). Notably, among all the 90 liver metastatic lesions, activity levels of EMT and angiogenesis were positively correlated with the immune score, stromal score, CD8+ T cells, fibroblasts, and anti-tumor immune signatures (Spearman correlation analysis, *p* < 0·01; **Table S8**; **Figure 2F**). Similar differences between dHGP and rHGP liver metastatic lesions were further confirmed in two impure patients (**Figure S1**).

### Comparison of the transcriptome landscapes of primary lesions between 20 dHGP and 15 rHGP patients

**Figure 3:**
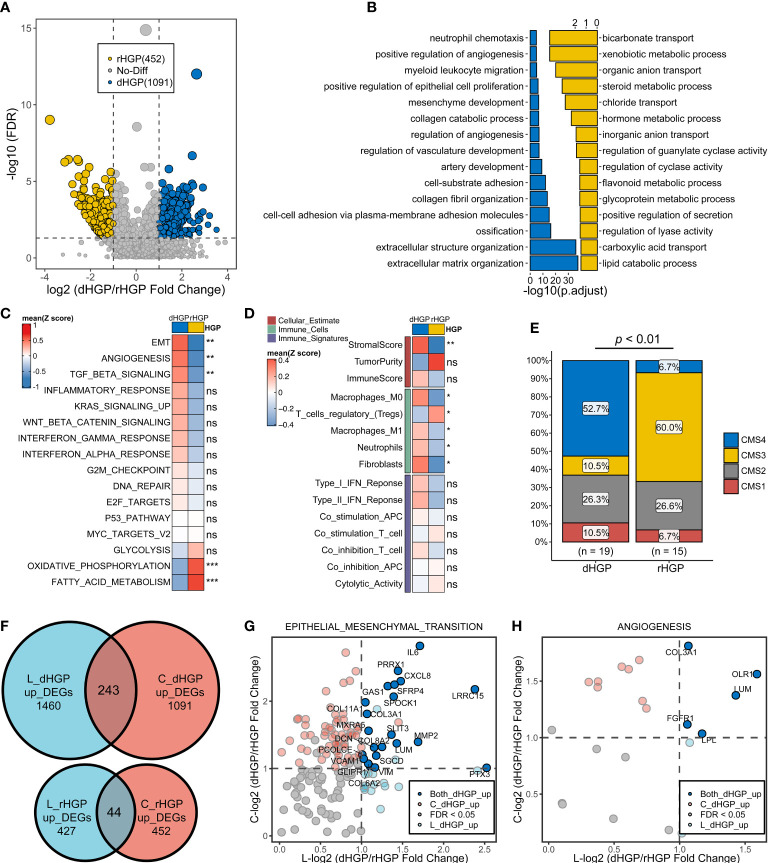
Comparison of the transcriptome landscapes of primary lesions between 20 dHGP and 15 rHGP patients. **(A)** Volcano plot displaying 1091 up-regulated DEGs in the dHGP subgroup and up-regulated 452 DEGs in the rHGP subgroup (|LogFC| > 1, FDR < 0·05). **(B)** GO-BP enrichment analysis of the differentially expressed genes. **(C)** Heatmap depicting the differences of HALLMARK pathways activity between dHGP and rHGP subgroups (Wilcoxon test. ns, *p* > 0·05; **p < 0·01; ***p < 0·001). Rows represent the mean GSVA scores of HALLMARK pathways, and columns represent HGP subgroups. **(D)** Heatmap depicting the differences between two subgroups in TIME-relevant molecular signatures were linked to cellular estimates, immune cells and immune signatures (Wilcoxon test. ns, *p* > 0·05; **p < 0·01). Rows represent mean ssgsea scores of TIME-relevant molecular signatures, and columns represent HGP subgroups. **(E)** Proportion of different CMS subtypes of primary lesions in dHGP or rHGP subgroups (Chi-square test, *p* < 0·01). **(F)** Venn diagram illustrating the intersection of DEGs in dHGP and rHGP subgroups respectively between the primary lesions and liver metastases. **(G)** Volcano plot showing the common up-regulated DEGs in the EMT pathways in the dHGP subgroup between primary lesions and liver metastases. **(H)** Volcano plot showing the intersection of DEGs up-regulated in angiogenesis pathway in the dHGP subgroup between primary lesions and liver metastases. *p < 0.05.

Analysis of the primary lesions showed that 1091 DEGs were up-regulated in dHGP subgroup and 452 DEGs were up-regulated in the rHGP subgroup (|LogFC > 1|, FDR < 0·05; **Table S10**; **Figure 3A**). GO-BP enrichment analysis of 1091 DEGs (FDR < 0·05; **Table S11**; **Figure 3B**) and HALLMARK pathways analysis (Wilcoxon test, *p* < 0·05; **Tables S4, S5**; **Figure 3C**) revealed that the primary lesions of dHGP patients were associated with EMT, angiogenesis, and transforming growth factor-β (TGF-β). Interestingly, primary lesions of rHGP patients were also enriched in metabolism-related pathways, including oxidative phosphorylation and fatty acid metabolism.

Unlike metastatic liver lesions, dHGP patients showed a higher stromal score but not immune score in primary lesions. A higher abundance of fibroblasts, M2 macrophages, M0 macrophages and neutrophils were present in primary lesions of dHGP patients (Wilcoxon test, *p* < 0·05; **Tables S6, S7**; **Figure 3D**). Primary lesions of rHGP patients exhibited a higher abundance of regulatory T cells. Consensus molecular subtypes (CMS)were different in two types of patients. Primary lesions of dHGP patients were characterized by CMS4 while rHGP patients were marked with CMS3 (**Table S12**; Chi-square test, *p* < 0·01).

The common DEGs between primary and metastatic liver lesions included 243 up-regulated genes in the dHGP subgroup and 44 up-regulated genes in the rHGP subgroup (**Figure 3F**). Both primary and metastatic lesions in the dHGP subgroup showed enrichment in the EMT and angiogenesis pathways (**Table S13**). As shown in **Figures 3G, H**, we summarized the intersecting up-regulated DEGs between the primary and metastatic liver lesions of the dHGP subgroup that were also enriched in the EMT and angiogenesis pathways.

### Comparison of the transcriptome landscapes of normal liver tissues between 20 dHGP and 15 rHGP patients

**Figure 4:**
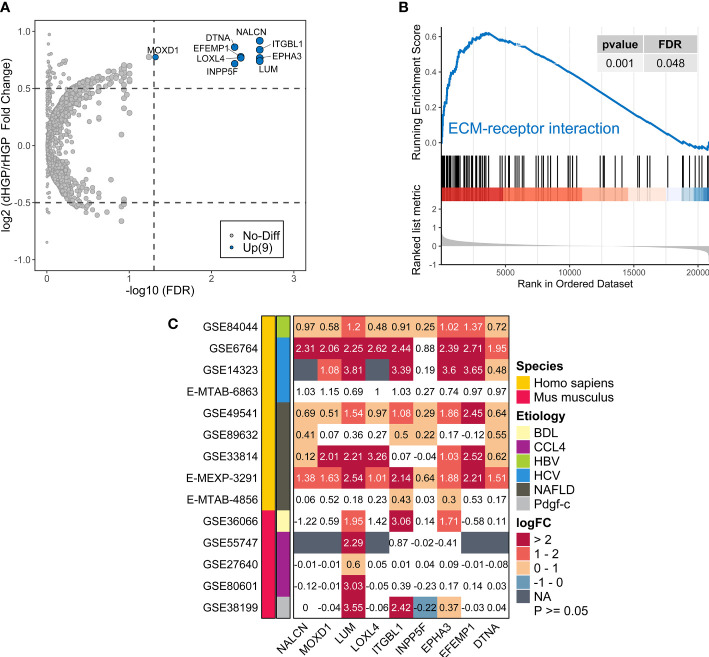
Comparison of the transcriptome landscapes of normal liver tissues between 20 dHGP and 15 rHGP patients. **(A)** Volcano plot showing 9 upregulated DEGs in the dHGP subgroup (|LogFC| > 0·5, FDR < 0·05). **(B)** Enrichment plots showing ECM receptor interaction pathways in the dHGP group. **(C)** Heatmap depicting the differential expression of 9 DEGs in the fibrosis or cirrhosis group versus the normal group in 14 public datasets of fibrotic livers caused by non-cancerous diseases. Numbers in each square box represent the fold change. NAFLD indicates nonalcoholic fatty liver disease. HCV indicates the hepatitis C virus. HBV indicates the hepatitis B virus. BDL indicates bile duct ligation. CCL4 indicates carbon tetrachloride. Pdgf indicates platelet-derived growth factor **(C)** BDL, CCL4, and Pdgf-c are the modeling methods of liver fibrosis in mice.

In normal liver tissues, 9 DEGs were up-regulated in dHGP patients but there was no DEGs in rHGP patients (|LogFC| > 0·5, FDR < 0·05; **Table S14**; **Figure 4A**). GO-BP enrichment analysis revealed that *LUM* was associated with the regulation of transforming growth factor beta1 production, which also participates in the process of collagen fibril organization along with *LOXL4*, *EPHA3* and *ITGBL1* were associated with cell-matrix adhesion. (FDR < 0·05; **Table S15**; **Figure S2A**). In addition, GSEA analysis of the KEGG pathways showed that normal liver tissues in the dHGP subgroup were enriched in the structural extracellular matrix (ECM)-receptor interaction (FDR < 0·05; **Table S16**; **Figure S2B** & **Figure 4B**). The above evidence prompted us to believe that the fibrosis progression of normal liver tissues might be implicated in the formation of different HGPs.

Furthermore, the differential expression of 9 DEGs in 14 transcriptome datasets of fibrotic livers caused by non-cancerous diseases was examined. Compared to the normal group, expression of all the 9 genes increased in the fibrotic or cirrhotic group of at least 4 human datasets. The expressions of 7 genes (*NALCN*, *MOXD1*, *LUM*, *ITGBL1*, *EPHA3*, *EFEMP1, DTNA*) were increased in 6 or more human datasets. The mRNA expression of 3 genes (*LUM*, *ITGBL1, EPHA3)* was markedly elevated in the fibrotic subgroup across humans and mice, regardless of the underlying etiology (LogFC > 0, FDR < 0·05; **Figure 4C**). However, the extent of mRNA expression fold change of 9 genes varied among different cohorts, which would require further validation. In summary, the expression of 9 DEGs was up-regulated with advancing fibrosis, while there were only modest changes in normal situations or mild fibrosis. Therefore, we surmised that normal liver tissue fibrosis might contribute to different HGP-type formations in liver metastases.

### The hypothesis of the formation of different HGPs formation in metastatic liver lesions

To provide a biological interpretation of the different HGP types, we constructed two scoring systems to quantify the differences between the primary lesions (C) and normal liver tissues (Ln) of dHGP and rHGP patients.

As shown in **Figure S3A**, the common DEGs between primary lesions and liver metastases included 243 up-regulated genes in the dHGP subgroup and 44 up-regulated genes in the rHGP subgroup. The scores of these up-regulated genes were quantified by the “ssGSEA” method (15) and were defined as dHGP score and rHGP score, respectively. An approach similar to “Gene expression grade index” (16) was utilized to calculate the C-score of each pure patient: C-score = dHGP score - rHGP score, which represented each patient’s transcriptomic characterization of the intrinsic inheritance of the primary lesion respectively. Similarly, 9 up-regulated DEGs in the normal liver tissues of the dHGP subgroup were quantified and considered as the Ln-score, which represented individual transcriptomic characterization of the normal liver microenvironment during the formation of liver metastases from primary tumor cells. Combined with the previous findings, the Ln-score indirectly reflected the degree of fibrosis in normal liver tissues.

**Figure 5:**
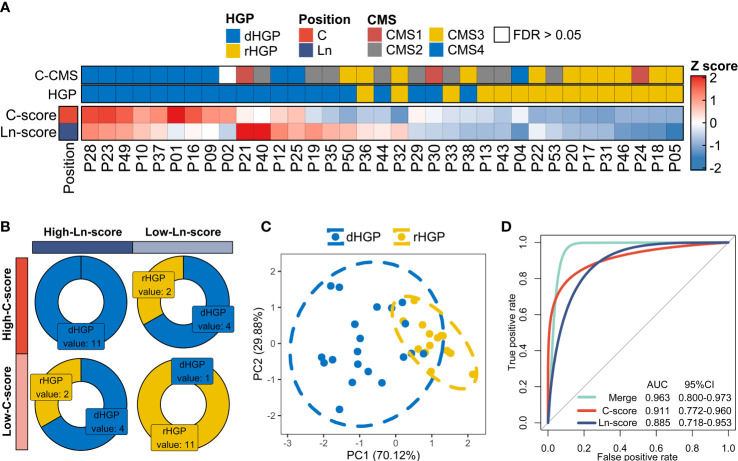
Exploration in the hypothesis of the formation of different HGPs in liver metastases. **(A)** Hierarchical clustering of individual C-score and Ln-score to classify 20 dHGP and 15 dHGP patients. “Euclidean” method was applied to calculate the distance. Column dendrograms were hide. Heatmap showing CMS subtypes of each patient. **(B)** Distribution of dHGP and rHGP patients stratified by both C-score and Ln-score (Chi-square test, *p* < 0·01). The two scores were divided into high and low groups based on the median. **(C)** PCA plot of patients in different HGP subgroups based on the individual C-score and Ln-score. **(D)** The predictive values for HGP types of the C-score, Ln-score, and both were measured by ROC curves among 35 patients.

Compared to the rHGP patients, dHGP patients showed a much higher C-score and Ln-score (*p* < 0·01; **Table S18**; **Figures S3B, S3C**). In other words, the higher the C-score or Ln-score, the more likely that the liver metastatic lesions possessed a desmoplastic growth pattern. This was further confirmed by the hierarchical clustering of individual C-score and Ln-score, which classified 20 dHGP and 15 dHGP patients (**Figure 5A**). 11 dHGP patients (100%, 11/11) displayed both a high C-score and a high Ln-score while 11 rHGP patients (91.7%, 11/12) were found to be associated with a low C-score and a low Ln-score (Chi-square test, *p* < 0·01; **Figure 5B**). In addition, 8 dHGP patients (75%, 8/12) were shown to be either associated with a high C-score or a high Ln-score. Patients with different HGPs were divided into separate groups based on the two scores, further confirmed by PCA analysis (**Figure 5C**). Moreover, the AUC of the combination of C-score and Ln-score measured by ROC curves among the patients was 0.963 (95%CI: 0·800-0·973), which was higher than the AUC of either one independently (**Figure 5D**).

However, there were some exceptions; P38 was a dHGP patient who obtained a low C-score and Ln-score, which prompted us to believe that the factors influencing the formation of HGPs are more complex than the hypothesis we proposed in this study.

### Exploration of the predictive value of hepatic metastasis HGP type and transcriptome subtype for the survival of CRLM patients

Specimens from 41 patients were collected for this study. In addition to the 35 pure HGP patients, there were 6 impure HGP patients. Those impure patients and pure rHGP patients were defined as non-dHGP patients. One pure dHGP patient was lost to follow-up, and 40 patients (19 dHGP vs 21 non-dHGP) were included in this part.

**Figure 6:**
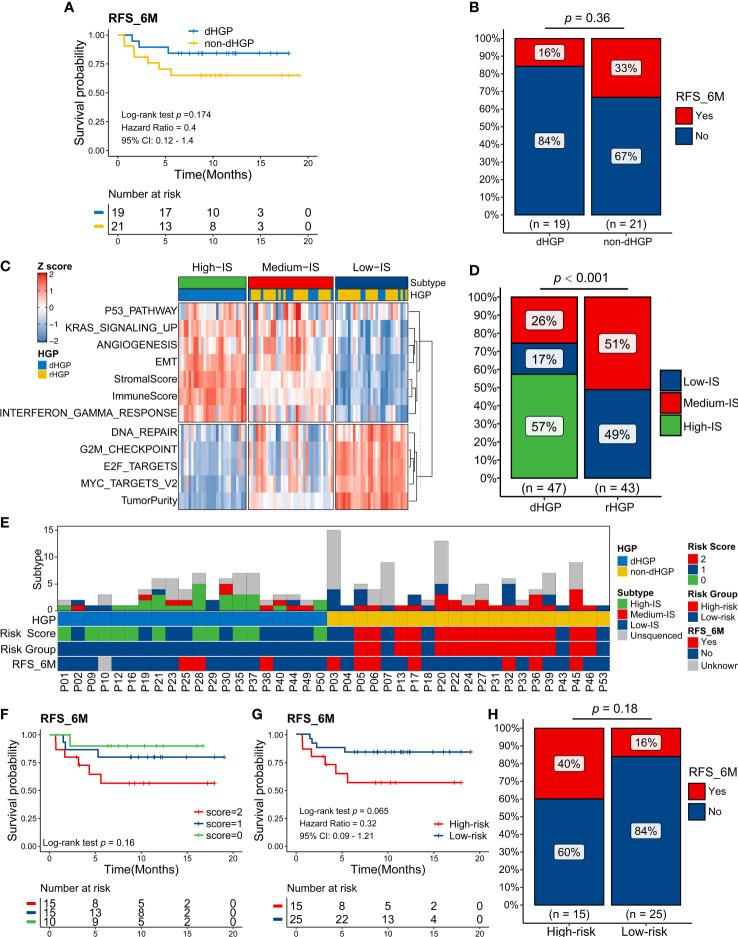
Exploration in the predictive value of the HGP type and transcriptome subtype of liver metastases for the survival of CRLM patients. **(A)** Kaplan-Meier curves for 6 months Relapse-Free Survival (RFS6M) of 19 dHGP patients and 21 non-dHGP patients (Log-rank test, *p* = 0.174). **(B)** Rate of early relapse events within 6 months (RFS_6M, Yes and No) in dHGP or non-dHGP subgroups (Chi-square test, *p* = 0.36). **(C)** Unsupervised clustering of important HALLMARK pathways and TIME-relevant molecular signatures among 90 liver metastases. Rows represent scores of various signatures, and columns represent samples. **(D)** Rate of three transcriptome subtypes in dHGP or non-dHGP subgroups (Chi-square test, *p <* 0·001). **(E)** Heatmap showing the transcriptome subtypes, HGP, risk score, risk subgroup, and early relapse event within 6 months of each patient. **(F)** Kaplan-Meier curves for RFS6M of patients with different risk scores (Log-rank test, *p* = 0.16). **(G)** Kaplan-Meier curves for RFS6M of patients with different risk subgroups (Log-rank test, *p* = 0.065). **(H)** Rate of early recurrence events within 6 months (RFS_6M, Yes and No) in high-risk or low-risk subgroups (Chi-square test, *p* = 0.18).

In contrast to non-dHGP patients, dHGP patients were more likely to have a better 6 months RFS (RFS6M) (Log-rank test, *p* = 0·174, HR: 0.4 (95%CI: 0·2 to 1·4), **Figure 6A**; Chi-square test, *p* = 0·36, **Figure 6B**). Previous studies suggested that integrative molecular subtyping of the liver metastases could determine the prognosis of CRLM patients (17, 18). Based on the HALLMARK pathways and TIME-relevant molecular signatures, we performed unsupervised clustering to classify 90 liver metastases into three biologically distinct transcriptome subtypes, termed as High-IS (immune score and stromal score), Medium-IS and Low-IS (**Figure S4**; **Figure 6C**). Significant differences were observed in the distribution of three transcriptome subtypes in dHGP or rHGP metastatic lesions (Chi-square test, *p <* 0·001; **Figure 6D**). Notably, rHGP metastases displayed no High-IS metastases subtype and more Medium-IS and Low-IS metastases subtypes than dHGP metastases.

However, in some metastatic lesions, the same HGP contains different transcriptomic isoforms, suggesting heterogeneity at the molecular level. Considering that the HGP type and transcriptome subtype contribute to patients’ prognosis, we defined a novel risk score based on them and classified patients into two main groups (**Figure 6E**). The non-dHGP and transcriptome subtype with Medium-IS subtype were assigned 1 point, while the dHGP and transcriptome subtype without Medium-IS subtype were assigned 0 points. Patients with 0-1 points in total were defined as low-risk, while those with 2 points were considered high risk.

Kaplan-Meier curves for RFS6M patients with different risk scores showed that risk score positively correlated with early recurrence (Log-rank test, *p* = 0·16; **Figure 6F**). Furthermore, the patients in high-risk subgroup displayed shorter RFS6M than patients in low-risk subgroup (Log-rank test, *p* = 0·065, HR: 0.32 (0·09-1·21); **Figure 6G**). Sixteen percent of low-risk and 40% of high-risk patients developed recurrence within 6 months after hepatectomy (Chi-square test, *p* = 0·18; **Figure 6H**).

## Discussion

To our knowledge, this is the first study that performed comprehensive analyses and comparisons at the RNA level on primary tumors, liver metastases and normal liver tissues, using two HGP subtypes in the CRLM. The mainly comparison of the transcriptome landscapes between the dHGP and rHGP were summarized in **Table 2**. We found that desmoplastic and replacement liver metastases showed different dysregulated mechanisms at the RNA level, mostly matching histopathological morphologies. The different expression genes in liver metastases might be partly inherited from the primary tumors, and the normal liver parenchymal seemed to possess different inflammatory microenvironments between the two HGP subtypes.

**Table 2:** The mainly comparison of the transcriptome landscapes between the dHGP and rHGP.

	dHGP vs rHGPin metastatic liver lesions (n = 47 vs n = 43)	dHGP vs rHGPin primary colorectal lesions (N = 20 vs N = 15)	dHGP vs rHGPin normal liver tissues (N = 20 vs N = 15)
**Number of DEGs**			
up-regulated in the dHGP	1460 (77.3%)	1091 (70.7%)	9 (100%)
up-regulated in the rHGP	427 (22.6%)	452 (29.3%)	0 (0%)
**Gene sets analyses**			
Inflammatory response	↑	↑	–
EMT	↑	↑	–
Angiogenesis	↑	↑	–
DNA repair	↓	–	–
Fatty acid metabolism	↓	↓	–
Oxidative phosphorylation	↓	↓	–
G2M_checkpoint	↓	–	–
KRAS signaling	↑	–	–
TGF-β signaling	–	↑	–
ECM-receptor interaction	/	/	↑
**Cell types**			
Tumor purity	↓	–	–
Immunes core	↑	–	–
Stromal score	↑	↑	–
Macrophage	↑	↑	–
Fibroblast	↑	↑	–
CD8+ T cell	↑	–	–
B cell	↑	–	–
**CMS**			
CMS 1	/	2 (10.5%) vs 1 (6.7%)	/
CMS 2	/	5 (26.3%) vs 4 (26.6%)	/
CMS 3	/	2 (10.5%) vs 9 (60.0%)	/
CMS 4	/	10 (52.7%) vs 1 (6.7%)	/

Noting: ↑: up-regulated in the dHGP; ↓: up-regulated in the rHGP;/, no analysis; -, no significance. HGP, histopathological growth pattern; dHGP, desmoplastic histopathological growth pattern; rHGP, replacement histopathological growth pattern; DEGs, differential expression genes; CMS, consensus molecular subtypes.

Higher inflammatory and immune responses were exhibited in the dHGP at the RNA level, consistent with previous studies showing that dHGP correlated with increased cytotoxic immune infiltrate in the immunohistochemistry and flow cytometry (19). This might explain the superior survival of patients with dHGP. In contrast, the rHGP seemed to be good at proliferation and DNA damage repair at the RNA level, which was not directly shown in morphology. Correspondingly, rHGP lesions showed more aggressive invasiveness and metastasis potential. Thus, histopathological variations are observable morphological changes resulting from the regulation of molecular expressions, and RNA sequencing could provide more details about the HGP features.

We found that the primary tumors with dHGP liver metastases also showed upregulation of EMT, angiogenesis, and the TGF-β signal pathway, which indicated that many liver metastases signatures were inherited from their primary tumor. The tumor border configuration of primary CRC also displayed different growth patterns. The pattern circumscribed with clearly desmoplastic stromal was consistently associated with a favorable prognosis and might indicate specific gene changes (20). According to a previous study, liver metastases originating from breast cancers mostly showed a predominance of rHGP (21). Therefore, it is reasonable to presume that the primary tumor may be responsible for the HGP in metastases. However, there was no significant difference in immune infiltration between the two HGP types of primary tumors. Our previous study also found that there was no significant correlation between the immune status of primary tumors and liver metastases, and the immune score of the primary tumor could not predict the prognosis of CRLM (22).

The soil of the liver is a fertile and popular site for excessive and complicated immunological activities (23). Several specific molecules were up-regulated in the normal liver tissue of patients with dHGP, related to inflammations in non-neoplastic lesions of the human liver. However, these genes were not significantly up-regulated in mice models. This might be an important reason why few liver metastases exist the dHGP in diverse animal models of CRLM unless specific genetic modification (24). These results strongly suggest that the microenvironment of the liver plays an essential role in determining HGP.

**Figure 7:**
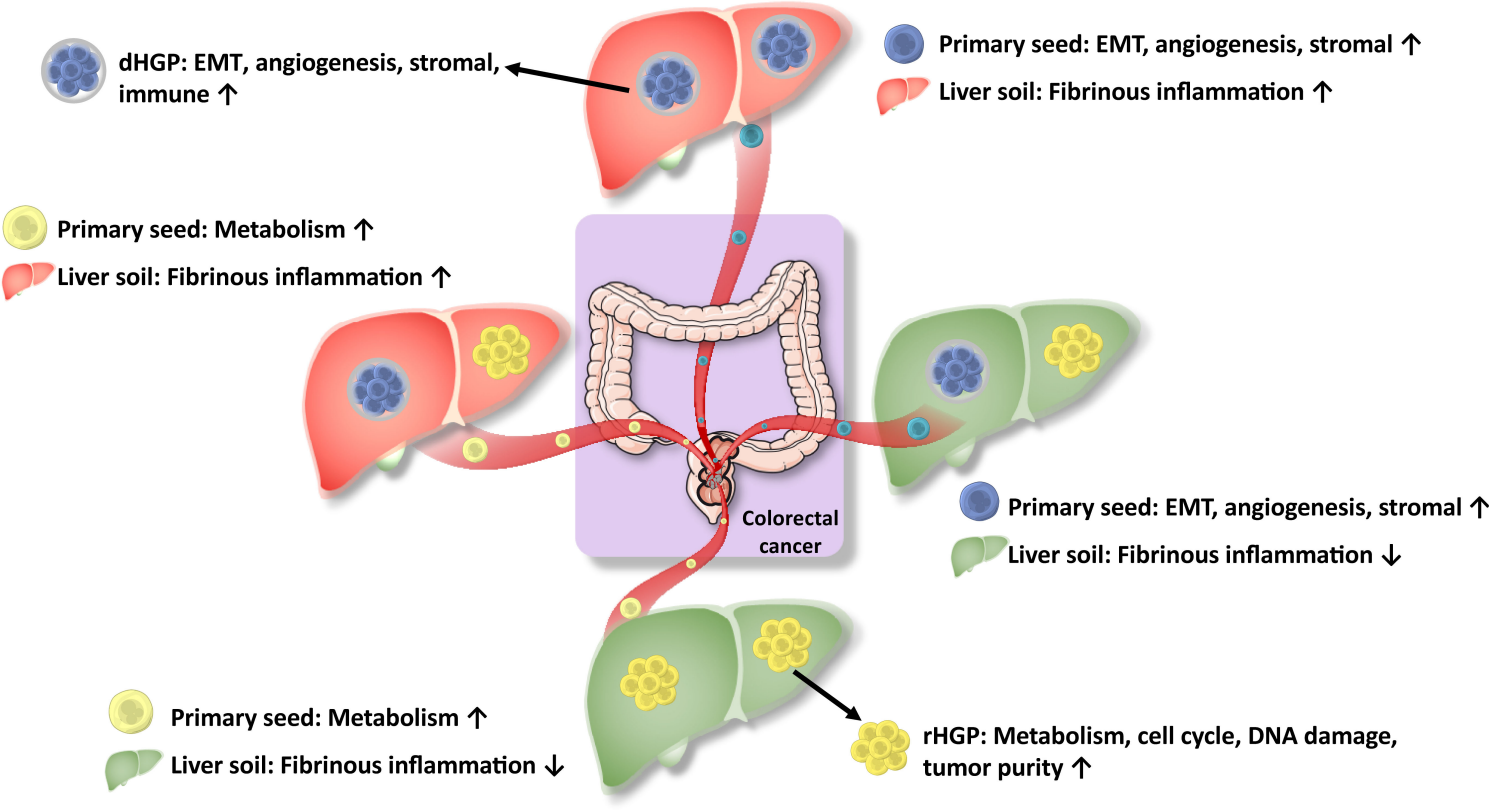
Hypothesis for regulation of molecular expressions for HGPs. HGPs are observable morphological changes resulting from the regulation of molecular expressions, which is the combined effect of the heterogeneity and remodeling of primary tumors seeds and liver soils.

The mechanism of HGPs formations is still unknown. The widely accepted “seed and soil” hypothesis described the metastases, which are the tumor cells as the “seeds” while the “suitable soil” is the metastatic microenvironment (25, 26). Thus, we speculated that the heterogeneity or reprogramming of seeds and soils might contribute to different HGPs (**Figure 7**). There may be two types of liver soil (one with increased inflammation and the other without) and two types of primary seeds (one with enhanced EMT and angiogenesis, and the other with enhanced metabolisms). If primary seeds with enhanced EMT and angiogenesis land on the inflammatory soil, they would probably result in desmoplastic lesions. In contrast, replacement lesions would arise when seeds with enhanced metabolisms fall into the non-inflammatory soil. And liver metastases would be very heterogeneous if seeds with enhanced EMT and angiogenesis go to the non-inflammatory soil or the inflammatory soil acquires seeds with enhanced metabolisms. However, we must admit that the hypothesis of the formation different HGPs is a bit far-fetched. For the C-score and Ln-score, only genes that were also significantly different in the lesions are included, which meant we seemed to know that they were different between the HGP’s in liver metastases. All data should be seen as hypothesis-generating and we need validation data to prove the hypothesis.

The CMS was developed based on abundant and independent gene expression data, but most patients were TNM I-III (27). In our cohort, most primary tumors with the dHGP liver metastases were classified as CMS4, while most rHGP showing metabolic dysregulation were classified as CMS3. In this study, we perform unsupervised clustering to classify liver metastases into three biologically distinct transcriptome subtypes. The contribution of different molecular features was similar to a previous study on curable oligometastatic CRLM (17). Subtype Low-IS metastases displayed enrichment for expression patterns associated with low immune and stromal infiltration. However, they were markedly enriched in E2F/MYC pathways and abnormalities in cell cycle checkpoints and DNA repair signaling, similar to the canonical subtype described in the previous study (17). In addition, High-IS and Medium-IS metastases subtypes were enriched for EMT, angiogenesis, and KRAS signaling. However, the High-IS metastases subtype showed a higher immune score than the Medium-IS metastases subtype. Therefore, the High-IS metastases subtype was more similar to the immune subtype as mentioned in the previous study, while Medium-IS metastases were more similar to the stromal subtype (17).

In this study, we provided a comprehensive molecular pathological subtype for patients with CRLM, in which the patients possessing the dHGP subtype and overexpressed immune genes showed a low risk of early recurrence. Although pathological morphology and molecular phenotype were consistent, the combination still showed better predictive power, which might guide treatment strategy, including targeted therapy and immunotherapy. However, more sample sizes and external validation are needed to verify the accuracy of our model.

Nevertheless, our study has some limitations. Firstly, most patients in our cohort received preoperative systemic chemotherapy leading to potential confounding effects on the HGP of CRLM and remodeling of the immune microenvironment (28, 29). Some metastatic lesions were so sensitive to chemotherapy that large areas of tumor cells were necrotic and tumors even achieved pathological complete response, leading to HGP evaluation failure. Secondly, our sample size was not large enough, and no public data was available to verify the results so that the findings were mainly speculative and descriptive. The follow-up time of survival analysis was insufficient, and overall survival data were not available. Thirdly, we did not perform *in vivo* or *vitro* experiments to uncover the critical molecules involved in the mechanism of HGP formation. This is because there is no accepted animal model of HGP to date. In the future, single-cell transcriptome spatial atlas and multiplexed tissue imaging are warranted to verify our findings further. Fourth, Some details are not well grasped. How normal were the areas selected for RNA-seq actually, and how far were these areas from the liver metastases? An array of technologies that offer improved resolution, often at the single cell level, including multiplexed immunofluorescence, single-cell RNA sequencing, and spatial transcriptional profiling, are now available and would be more appropriate analysis approaches than bulk RNA sequencing, which is confounded by differing abundances of different cell populations. In the future, we could use these techniques to further investigate.

In conclusion, We uncovered that the histopathological growth patterns of liver metastases were observable morphological changes arising from the regulation of molecular expressions through the combined effect of the heterogeneity of primary tumors (the seeds) and liver remodeling (the soil) (**Figure 7**).

## Data availability statement

All the raw data was deposited into Research Data Deposit (https://www.researchdata.org.cn/) with RDD number RDDB2022810006, and will be made available on request by the authors without undue reservation. Further inquiries can be directed to the corresponding authors.

## Ethics statement

The studies involving human participants were reviewed and approved by Sun Yat-sen University cancer center’s medical ethics committee. Written informed consent for participation was not required for this study in accordance with the national legislation and the institutional requirements.

## Author contributions

YHL, MSC and ZMD were involved in designing study, supervising the work, and data interpretation. MTH, ZGC and DDH were involved in protocol development, data analysis, and wrote the original draft. SYX, and MTH evaluated the HGPs. DSW, XLZ, WPF, LW and YYC were involved in data collection and study design. DDH, YFY, BKL, XJW, ZHL, CG, LRL, PRD, ZZP and DSW contributed to sample collection and data collection. All authors contributed to the article and approved the submitted version.
