# Supplementary material for: Delineating the molecular landscape of different histopathological growth patterns in colorectal cancer liver metastases
Source: Front Immunol. 2022 Dec 16;13:1045329. doi: 10.3389/fimmu.2022.1045329 (PMC9800416; doi:10.3389/fimmu.2022.1045329)
Supplement: Supplementary file 1 [file Table_1.docx]

Supplementary Material

# Supplementary Methods

**Quality control, mapping, quantification of the raw data.**

Raw data of fastq format for each sample were assessed for quality using the FastQC tool (version 0.11.5). Raw reads were aligned to the GRCh38 primary genome assembly using Spliced Transcripts Alignment to a Reference (STAR) aligner (version 2.2.1). RSEM (version 1.3.0) was used to count the reads numbers mapped to each gene. Transcripts per kilobase million (TPM) of each gene were calculated based on the reads count mapped to this gene and the length of the gene. All downstream analyzes involving the transcriptome were performed with TPM data, except for differential analysis, which was done with count data.

**Differential expression analysis and functional annotation of the differentially expressed genes (DEGs).**

Using the Deseq2 package for R^1^, we performed differential expression analysis between dHGP and rHGP subgroups among liver metastases, primary lesions, and normal liver tissues. A shrinkage estimator was implemented to test the distribution's variance, and the *p*-values were controlled for false discovery rate (FDR) by the Benjamini–Hochberg (BH) adjustment. The genes with absolute log2 fold-change > 1 and FDR < 0.05 were considered as differentially expressed genes (DEGs). DEGs were annotated by enrichment analysis of Gene Ontology biological process (GO-BP) using R package clusterProfiler.^2^ The BH method was applied to adjust the p-values and FDR < 0.05 was considered statistically significant. Gene Set Enrichment Analysis (GSEA) analysis of Kyoto Encyclopedia of Genes and Genomes (KEGG) pathways was performed by employing the R package clusterProfiler. All transcripts were ranked by log2 fold-change between the dHGP and rHGP subgroups. FDR < 0.25 was considered statistically significant.

**Logistic regression models for predicting HGPs of liver metastases and Gaussian mixture.**

To reduce the number of genes for the logistic regression model, 1887 DEGs were sorted by AUC to predict the HGPs of liver metastases, and 10 genes with the relative biggest AUCs were selected to align and perform 1,023 combinations analysis. For each combination, a logistic regression model was constructed, and a total of 1023 AUCs were generated. The clusters of 1023 models were classified by the Gaussian mixture model.^3^

**HALLMARK pathways and tumor immune microenvironment (TIME) relevant molecular signatures.**

To assign activity estimates of 50 HALLMARK pathways^4^ to each tumor sample, we employed [gene set variation analysis](https://pubmed.ncbi.nlm.nih.gov/23323831/) (GSVA),^5^ using the GSVA package in R. We selected 3 cellular estimates^6^, 23 immune cells^7, 8^ and 7 immune signatures^9^ as TIME-relevant molecular signatures. Except for fibroblast, which was calculated by MCPcounter, the other 22 immune cells were quantified by CIBERSORT. The R package IOBR^10^ was applied to quantify the scores of TIME-relevant molecular signatures. In addition, we collected 72 immune-related genes from Thorsson et al.^11^

**Consensus molecular subtypes (CMS) of primary lesions.**

To systematically elucidate the potential differences between dHGP and rHGP in primary lesions, we divided primary lesions into four distinct CMS by applying the "CMScaller" R package .^12^

**Exploration in the transcriptome datasets of fibrotic livers caused by non-cancerous diseases.**

To elucidate the potential influence of normal liver tissues on the formation of different HGPs, we explored the DEGs generated by Ln in the transcriptome datasets of fibrotic livers caused by various non-cancerous diseases. 14 public datasets were collected from the GEO database (https://www.ncbi.nlm.nih.gov/geo/) and the ArrayExpress database (https://www.ebi.ac.uk/arrayexpress), including 9 datasets from homo sapiens and 5 datasets from mus musculus. Detailed information of each dataset was summarized in Table S17. Samples of each dataset were divided into the fibrotic or cirrhotic group or the normal group, and differential expression analysis was implemented using the limma R package. ^13^

**Construction of the Ln-score and C-score.**

As shown in Fig S4A, the intersection of DEGs between primary lesions and liver metastases included 243 up-regulated genes in the dHGP subgroup and 44 up-regulated genes in the rHGP subgroup. Scores of 243 genes and 44 genes quantified by the "ssGSEA" method^5^ were defined as dHGP score and rHGP score, respectively. An approach similar to "Gene expression grade index" ^14^ was utilized to calculate the C-score of each pure patient: C-score = dHGP score - rHGP score, which represented each patient's transcriptomic characterization of the intrinsic inheritance of the primary lesion respectively. Similarly, 9 up-regulated DEGs in the dHGP subgroup of normal liver tissues were quantified and considered as the Ln-score, which represented individual transcriptomic characterization of the normal liver microenvironment during the formation of liver metastases from primary tumor cells.

**Transcriptome subtype of liver metastases.**

Based on the selected signatures of HALLMARK and TIME^15^, unsupervised clustering was applied to classify 90 liver metastases into three distinct transcriptome subtypes, termed as High-IS (immune score and stromal score), Medium-IS, and Low-IS. Using ConsensuClusterPlus package for R (Consensus Clustering: A Resampling-Based Method for Class Discovery and Visualization of Gene Expression Microarray Data), this process was executed with parameters including Euclidean distance and Ward's linkage and 1000 times repetitions.

# Supplementary References

1 Love MI, Huber W, Anders S. Moderated estimation of fold change and dispersion for RNA-seq data with DESeq2. Genome biology 2014; 15(12): 550.

2 Yu G, Wang LG, Han Y, He QY. clusterProfiler: an R package for comparing biological themes among gene clusters. Omics : a journal of integrative biology 2012; 16(5): 284-7.

3 Hong HC, Chuang CH, Huang WC, et al. A panel of eight microRNAs is a good predictive parameter for triple-negative breast cancer relapse. Theranostics 2020; 10(19): 8771-89.

4 Subramanian A, Tamayo P, Mootha VK, et al. Gene set enrichment analysis: a knowledge-based approach for interpreting genome-wide expression profiles. Proceedings of the National Academy of Sciences of the United States of America 2005; 102(43): 15545-50.

5 Hänzelmann S, Castelo R, Guinney J. GSVA: gene set variation analysis for microarray and RNA-seq data. BMC bioinformatics 2013; 14: 7.

6 Yoshihara K, Shahmoradgoli M, Martínez E, et al. Inferring tumour purity and stromal and immune cell admixture from expression data. Nat Commun 2013; 4: 2612.

7 Newman AM, Liu CL, Green MR, et al. Robust enumeration of cell subsets from tissue expression profiles. Nature methods 2015; 12(5): 453-7.

8 Becht E, Giraldo NA, Lacroix L, et al. Estimating the population abundance of tissue-infiltrating immune and stromal cell populations using gene expression. Genome biology 2016; 17(1): 218.

9 Rooney MS, Shukla SA, Wu CJ, Getz G, Hacohen N. Molecular and genetic properties of tumors associated with local immune cytolytic activity. Cell 2015; 160(1-2): 48-61.

10 Zeng D, Ye Z, Shen R, et al. IOBR: Multi-Omics Immuno-Oncology Biological Research to Decode Tumor Microenvironment and Signatures. Frontiers in immunology 2021; 12: 687975.

11 Thorsson V, Gibbs DL, Brown SD, et al. The Immune Landscape of Cancer. Immunity 2018; 48(4): 812-30.e14.

12 Eide PW, Bruun J, Lothe RA, Sveen A. CMScaller: an R package for consensus molecular subtyping of colorectal cancer pre-clinical models. Scientific reports 2017; 7(1): 16618.

13 Ritchie ME, Phipson B, Wu D, et al. limma powers differential expression analyses for RNA-sequencing and microarray studies. Nucleic acids research 2015; 43(7): e47.

14 Sotiriou C, Wirapati P, Loi S, et al. Gene expression profiling in breast cancer: understanding the molecular basis of histologic grade to improve prognosis. J Natl Cancer Inst 2006; 98(4): 262-72.

15 Pitroda SP, Khodarev NN, Huang L, et al. Integrated molecular subtyping defines a curable oligometastatic state in colorectal liver metastasis. Nat Commun 2018; 9(1): 1793.

# Supplementary Figures

**Figure S1: Comparisons of the HALLMARK pathways and TIME-related signatures between dHGP and rHGP subgroups in two impure patients.**


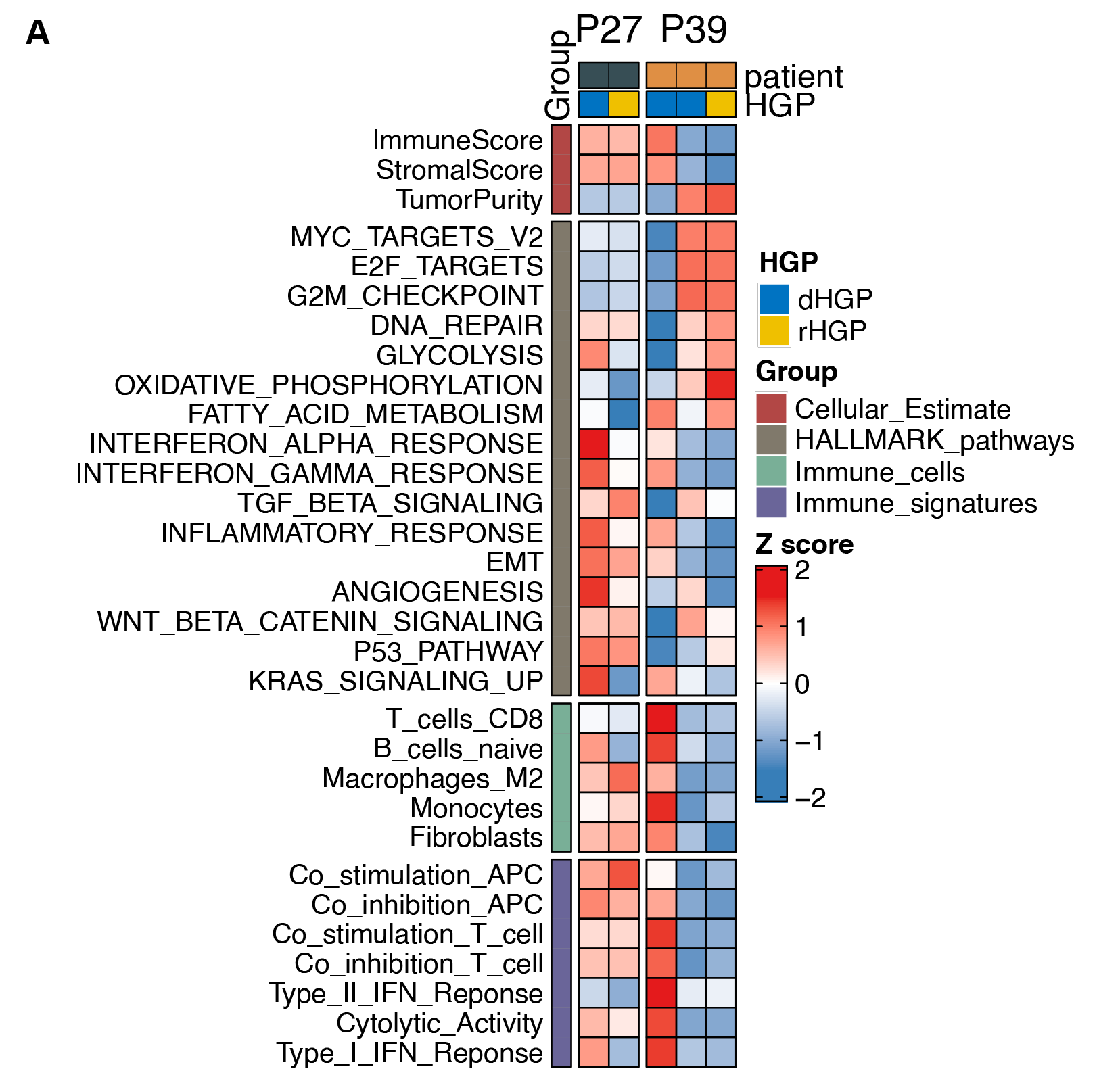


(A) Heatmap depicting the differences of HALLMARK pathways and TIME-related signatures between dHGP and rHGP lesions in two impure patients (P27 and P39).

**Figure S2: Functional enrichment analysis of DEGs between dHGP and rHGP subgroups in normal tissues (Ln) of metastatic liver lesions.**


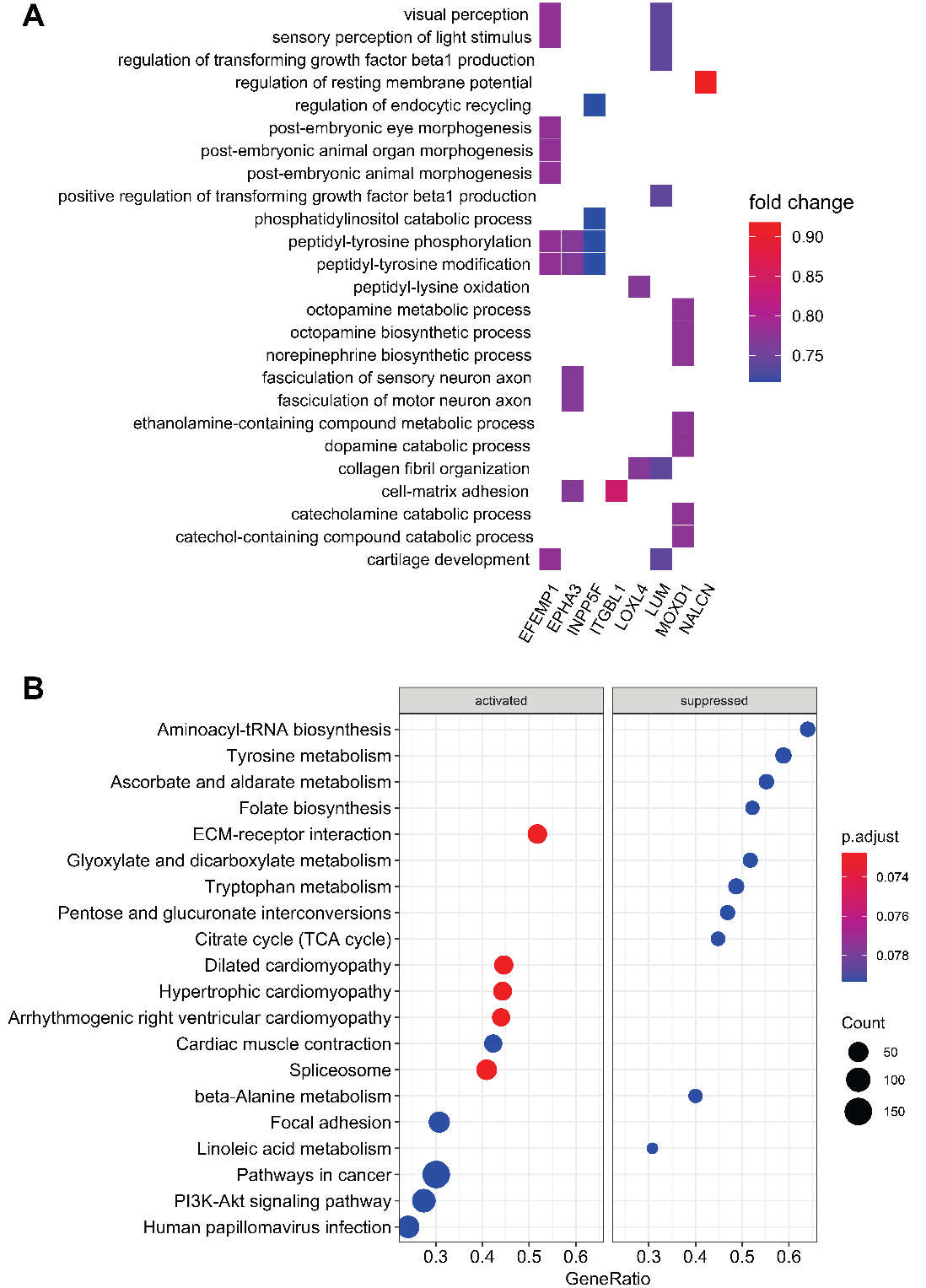


(A) GO-BP enrichment analysis of up-regulated genes in the dHGP subgroup of Ln.

(B) KEGG-GSEA analysis between dHGP and rHGP subgroups of Ln.

**Figure S3: Prediction of the HGP type of CRLM patients based on the C-score and Ln-score.**


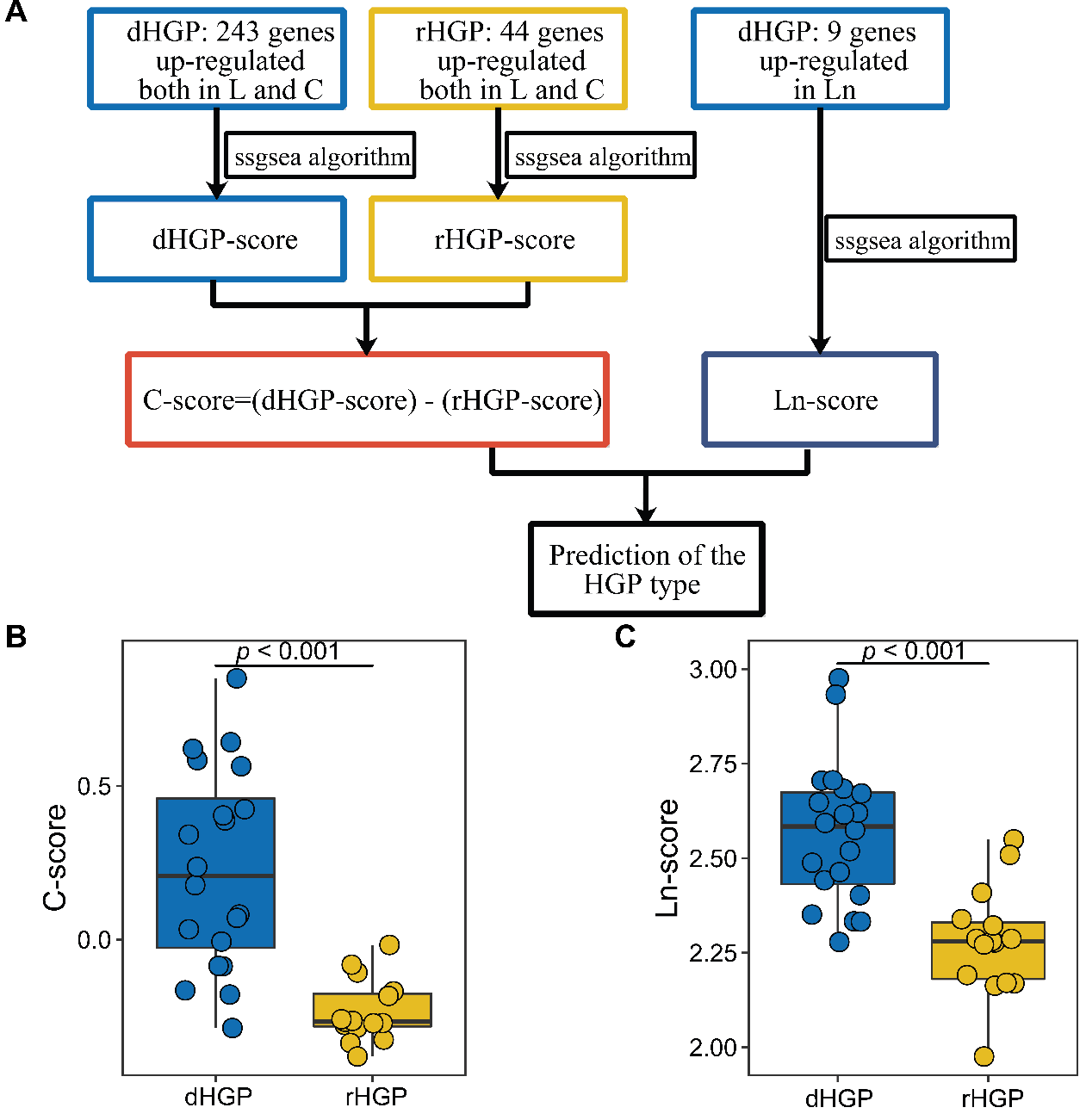


(A) Flow chart of the construction of C-score and Ln-score.

(B) Boxplot depicting the comparison of C-score between dHGP and rHGP subgroups.

(C) Boxplot depicting the comparison of Ln-score between dHGP and rHGP subgroups.

**Figure S4: Unsupervised clustering of 90 metastatic liver samples.**


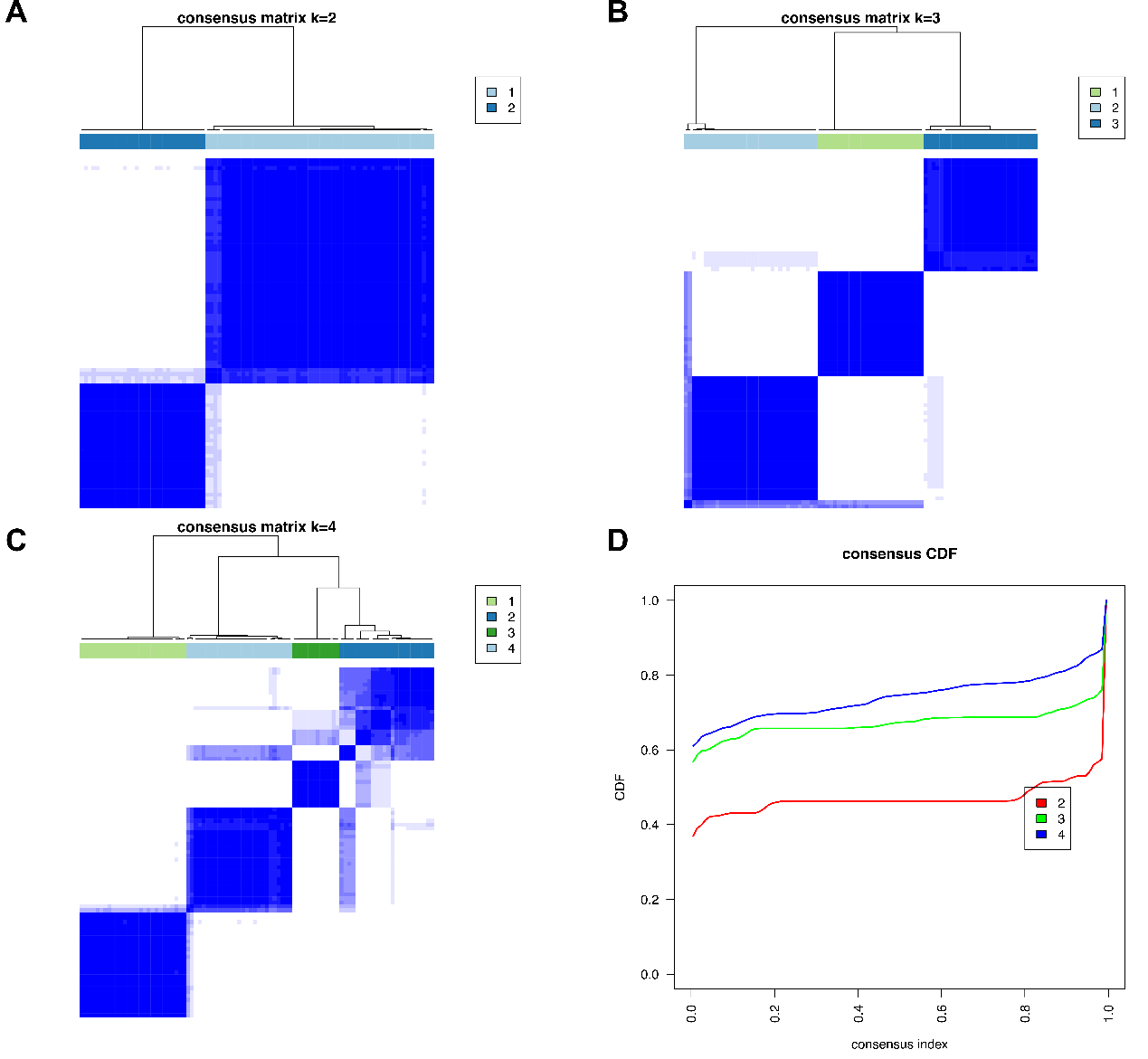


(A, B, C, D) Consensus matrixes of 47 dHGP and 43 rHGP metastatic liver samples for each k (k = 2–4) based on the HALLMARK pathways and TIME-related signatures, displaying the clustering stability using 1000 iterations of hierarchical clustering.
